# An artificial intelligent diagnostic system on mobile Android terminals for cholelithiasis by lightweight convolutional neural network

**DOI:** 10.1371/journal.pone.0221720

**Published:** 2019-09-12

**Authors:** Shanchen Pang, Shuo Wang, Alfonso Rodríguez-Patón, Pibao Li, Xun Wang

**Affiliations:** 1 College of Computer and Communication Engineering, China University of Petroleum, Qingdao, Shandong, China; 2 Departamento de Inteligencia Artificial, Universidad Politécnica de Madrid, Campus de Montegancedo, Boadilla del Monte, Madrid, Spain; 3 Department of Intensive Care Unit, Shandong Provincial Third Hospital, Jinan, Shandong, China; Polytechnical Universidad de Madrid, SPAIN

## Abstract

Artificial intelligence (AI) tools have been applied to diagnose or predict disease risk from medical images with recent data disclosure actions, but few of them are designed for mobile terminals due to the limited computational power and storage capacity of mobile devices. In this work, a novel AI diagnostic system is proposed for cholelithiasis recognition on mobile devices with Android platform. To this aim, a data set of CT images of cholelithiasis is firstly collected from The Third Hospital of Shandong Province, China, and then we technically use histogram equalization to preprocess these CT images. As results, a lightweight convolutional neural network is obtained in a constructive way to extract cholelith features and recognize gallstones. In terms of implementation, we compile Java and C++ to adapt to the application of deep learning algorithm on mobile devices with Android platform. Noted that, the training task is completed offline on PC, but cholelithiasis recognition tasks are performed on mobile terminals. We evaluate and compare the performance of our MobileNetV2 with MobileNetV1, Single Shot Detector (SSD), YOLOv2 and original SSD (with VGG-16) as feature extractors for object detection. It is achieved that our MobileNetV2 achieve similar accuracy rate, about 91% with the other four methods, but the number of parameters used is reduced from 36.1M (SSD 300, SSD512), 50.7M (Yolov2) and 5.1M (MobileNetV1) to 4.3M (MobileNetV2). The complete process on testing mobile devices, including Virtual machine, Xiaomi 7 and Htc One M8 can be controlled within 4 seconds in recognizing cholelithiasis as well as the degree of the disease.

## Introduction

Artificial intelligence (AI) systems have been applied to improve the delivery and effectiveness of health care [1–3]. Many of them are a triumph for science, representing years of improvements in computing power and the neural networks that underlie deep learning. AI diagnostic tools can find problems including retinal disease, but need to be developed with care. Particularly, AI medical image recognition tools have been increasingly concerned by the academic community and industry [4–6]. Some AI diagnostics tools have already found their way into clinical practice, but few of AI them are related to cholelithiasis recognition.

Nowadays, healthy information systems (HIS) can now provide digital diagnostic reports by APPs on Smart phones, but few AI diagnostics system are designed for mobile terminals due to the limited computational power and storage capacity of mobile devices. Recently, some multi-core parallel computing mobile devices chips have been developed, which can do the real-time processing of lower resolution images. In this circumstance, AI diagnose systems on mobile terminals have raised widely research interests [7–8].

In this work, we consider to developing AI diagnostics system for cholelithiasis recognition on mobile Android devices. To this aim, a dataset of cholelithiasis patients is crucially needed for training and verifying, since there is no open source data set of cholelithiasis and gallstones. It is collected the CT images of 100 patients with cholelithiasis from Shandong Provincial Third Hospital, which is confidential. After revolving the images, we obtain in total 1300 CT images of cholelithiasis, and 673 CT images are randomly selected for training and the rest 627 images are used for verification. To match the limited computational power and storage of mobile devices, we use lightweight convolution neural network and mobile terminal neural network model, see e.g. [9]–[14], to deploy a small deep convolution neural network model at the mobile terminal devices.

Data experimental results show that our system achieves an average accuracy of 90.8% in cholelithiasis recognition from CT images. We evaluate and compare the performance of our MobileNetV2 with MobileNetV1, Single Shot Detector (SSD) [15], YOLOv2 [16] and original SSD (with VGG-16) [17] as feature extractors for object detection. It is achieved that our MobileNetV2 achieve similar accuracy rate, about 91%, with MobileNetV1, SSD, YOLOv2 and original SSD (with VGG-16) but the number of parameters used is reduced from 36.1M (SSD 300, SSD512), 50.7M (Yolov2) and 5.1M (MobileNetV1) to 4.3M (MobileNetV2). The complete process on testing mobile devices can be controlled within 4 seconds in recognizing cholelithiasis.

## Our method

**Fig 1:**
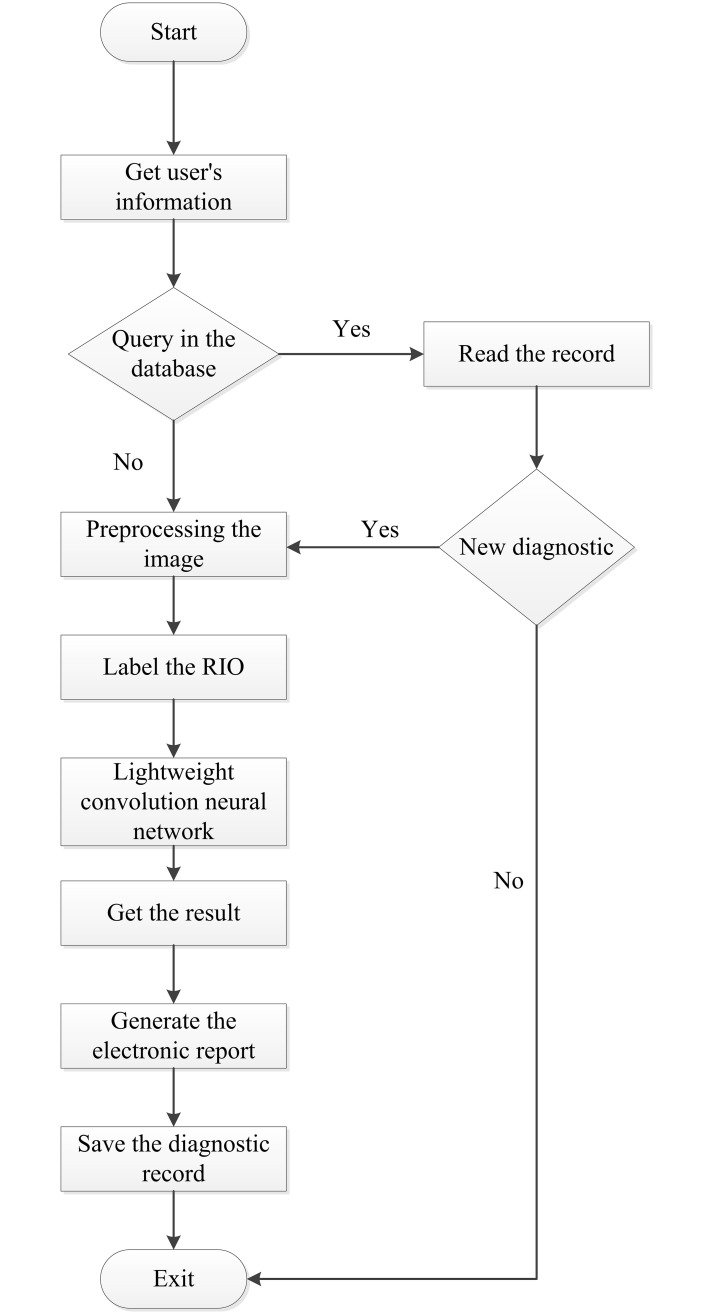
The flowchart of recognizing cholelithiasis.

Our AI diagnostic system on mobile devices for cholelithiasis has two constituent parts, which are image preprocessing and cholelithiasis recognizing parts. In image preprocessing part, medical image of cholelithiasis is taken as input. The contrast of the image is increased by histogram equalization, nonlinear stretching on the image, redistributing image pixel values, and making the number of pixels within a certain range of gray scale values roughly the same. Specifically, histogram equalization is used to balance the images that are too bright or dark in the background and foreground. Such method can display effectively the location of lesions in CT images of cholelithiasis. This completes the preprocess of CT medical images of cholelithiasis, which is the basis for the recognition of cholelithiasis. The flowchart of recognizing cholelithiasis is shown in Fig 1.

**Step 1**. Obtaining the user’s basic identity information and the original CT image of gallstone disease;
**Step 2**. Determining whether the database contains the diagnostic record of user;
**Step 3**. Preprocessing the image with histogram equalization to increase the contrast of the image;
**Step 4**. Labelling the ROI in the CT image processed;
**Step 5**. Inputting the image with ROI into the lightweight convolution neural network trained for recognition;
**Step 6**. Outputting the results of recognizing cholelithiasis;
**Step 7**. Generating an electronic medical report;
**Step 8**. Saving the user’s diagnostic record.

### Preprocessing CT images

There are three stages for recognizing cholelithiasis from CT images by our AI diagnostic system, which are preprocessing CT images of cholelithiasis, labelling the ROI in the CT image processed and recognizing cholelithiasis.

The preprocessing CT images directly determines whether the next step can be carried out smoothly. In preprocessing CT images stage, it needs to increase the contrast of the image by histogram equalization, redistribute image pixel values, and make the number of pixels within a certain range of gray scale values roughly the same. In consideration of the accuracy and speed of the system, histogram equalization is used to enhance the contrast of CT images of cholelithiasis.

Histogram equalization form [18] is applied to enhance the contrast of CT images. The histogram equalization algorithm transforms the histogram of the original image as a substantially uniform distribution over the entire range of gray scale, thereby expanding the dynamic range of the pixel gray scale, thereby enhancing the contrast of the image. The steps of using histogram equalization algorithm are shown as follows:

Firstly, giving all gray scale levels of the original image *S*_*k*_ with *k* = 0, 1, …, *L* − 1, where *L* = 256 is gray scale, and then counting the pixels of each gray scale of the original image *n*_*k*_. After that, we can calculate the gray scale histogram by $P\left(S_{k}\right)=\frac{n_{k}}{n}$, *k* = 0, 1, …, *L* − 1, where *n* is the number of pixels of all, *n*_*k*_ is the number of pixels of *S*_*k*_.

We can then calculate the cumulative histogram of the original image by

$$\begin{array}{c} t_{k}=EH\left(S_{k}\right)=\sum_{i=0}^{k}\frac{n_{i}}{n}=\sum_{i=0}^{k}P_{S}S_{i}, \end{array}$$

with 0 ≤ *S*_*k*_ ≤ 1, *k* = 0, 1, …, *L* − 1 and *P*_*S*_ being the gray scale histogram of *S*_*k*_, and round it by function $U_{k}=int\left[\left(L-1\right)t_{k}+\frac{k}{N}\right]$. After determining the mapping by function *S*_*k*_ → *U*_*k*_, it is counted the number *n*_*k*_ of pixels of per gray scale in the new histogram *S*_*k*_. The histogram can be updated by $P\left(t_{k}\right)=\frac{n_{k}}{n}$.

**Fig 2:**
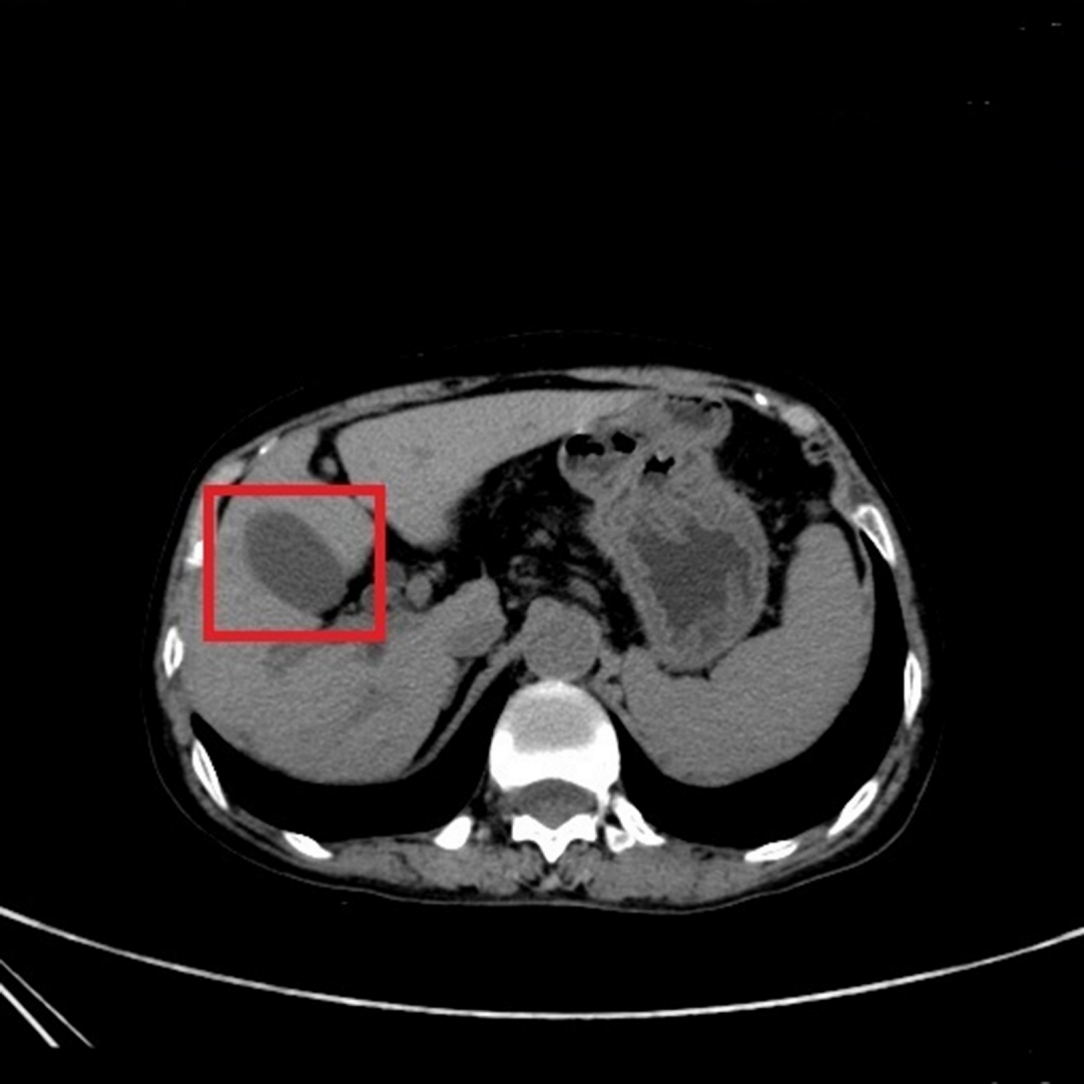
The CT image of a healthy person.

**Fig 3:**
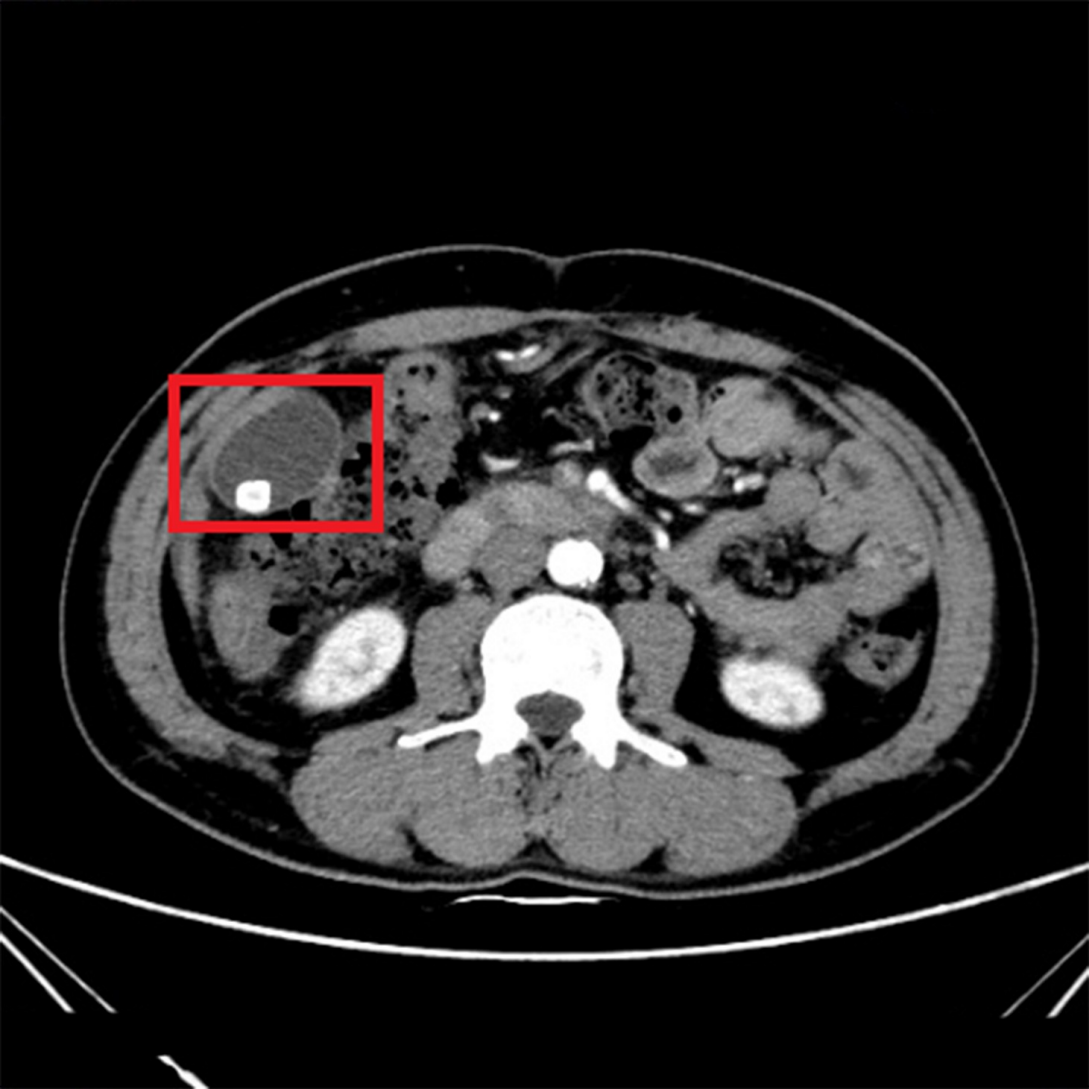
The original CT images of a person with the cholelithiasis.

**Fig 4:**
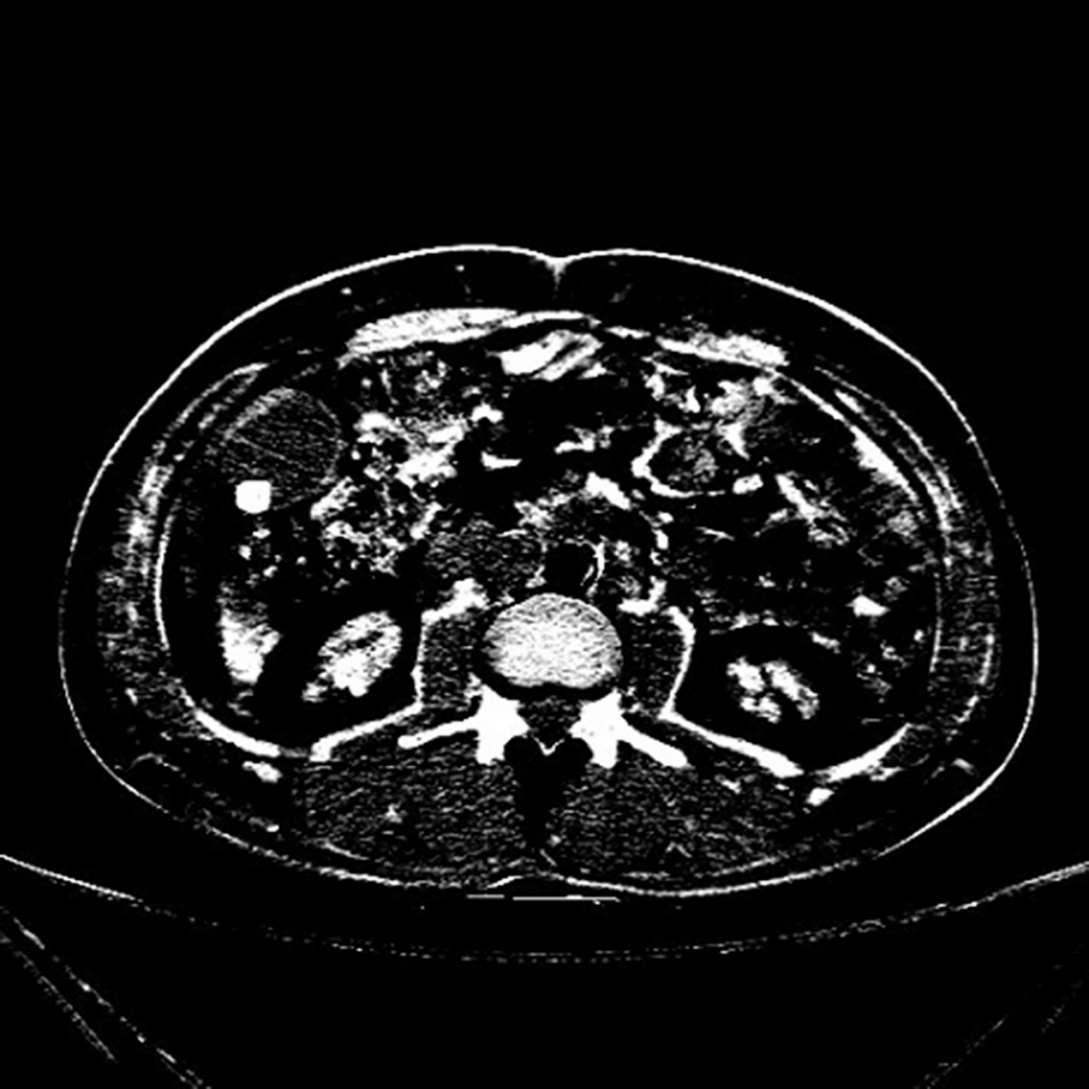
The CT image by histogram equalization.

In Figs 2 and 3, it shows CT images of a healthy person and the original CT images of a person with the cholelithiasis, respectively. It is not hard to find human tissues and organs in CT images, such as kidneys, gallbladder, spine, etc. We labeled the gallbladder in the picture with red boxes and it shows clearly the difference between the two pictures. In Fig 2, it is shown the gallbladder of healthy person is all light gray color to indicate that the gallbladder is healthy. However, in Fig 3, gallstones are white in color and differ significantly from the gallbladder in the CT images of a person with the cholelithiasis. In Fig 4, it gives the CT image processed by histogram equalization.

### Labelling ROI on CT images

Regions of importance (ROI) is regions of interest on CT images, whose labels are crucial for neural network training. We select an image region referred to as the ROI from the CT image, which is the focus of analysing image. Selecting the area that we want to get for further processing can reduce the processing time, increase the accuracy.

We input ROI into the lightweight convolution neural network trained for recognition instead of inputting the entire image processed histogram equalization. Most of the areas without cholelithiasis are filtered out by inputting ROI. The number of pixels in the ROI containing the lesions is smaller than the image was preprocessed only. The speed of recognition of cholelithiasis can be greatly improved. Meanwhile, the reduction of the number of pixels results in the reduction of the weight parameters of the neural network, and the utilization of the device memory will be decreased greatly. This makes it easier to deploy neural networks on mobile devices. We have completed manual operations to label the ROI on CT image for cholelithiasis recognition.

**Fig 5:**
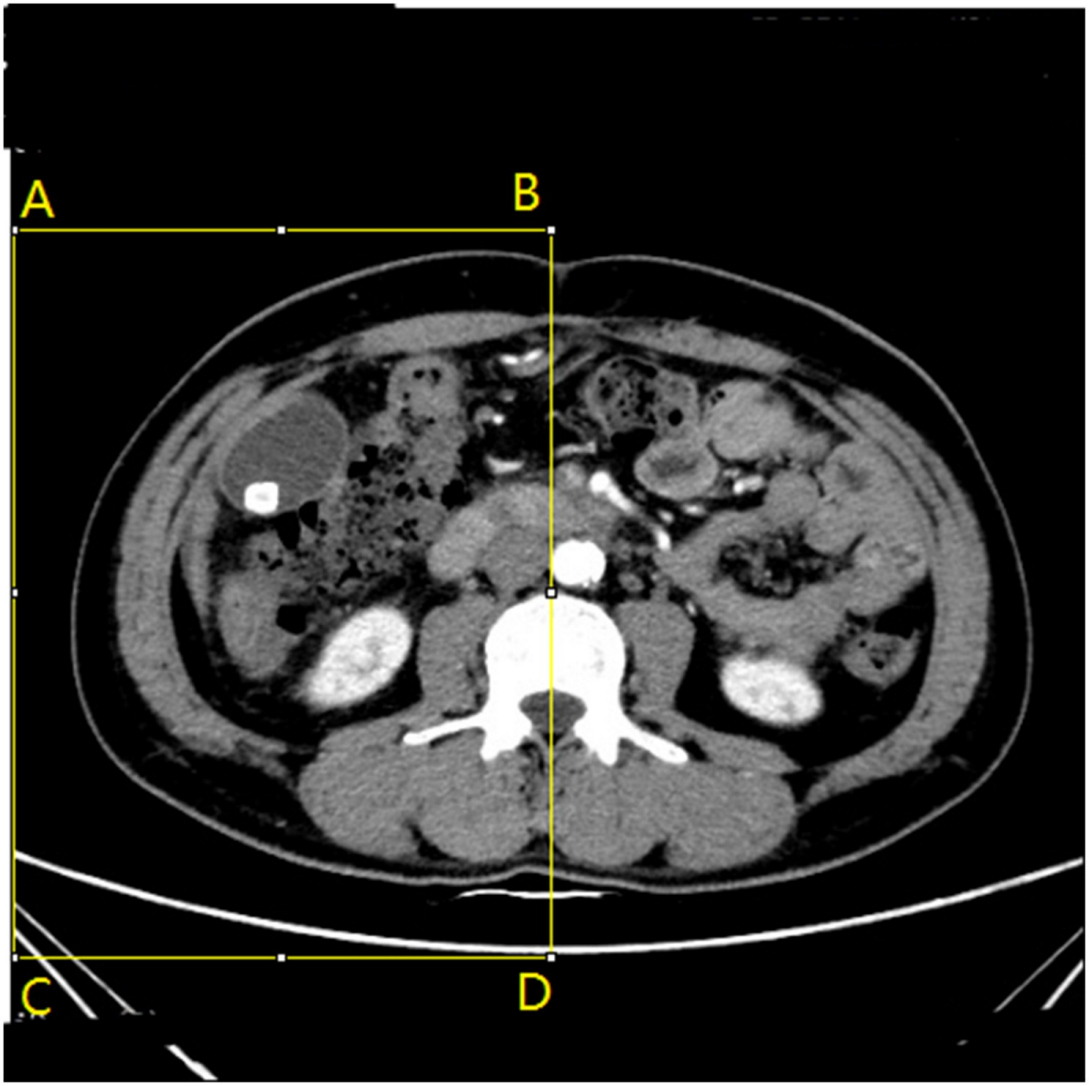
The CT image with ROI.

In order to improve the model generalization ability, the training data set was shifted, rotated and shrunk to enhance the training dataset. The enhanced training set in which the location of the gallstone lesions changes slightly. However, even with such changes in training set, the location of the gallstone lesions in all CT images remained in the left half. In addition, there is no any features in the some areas in the original CT images, and the areas in these images is useless. We set the ROI as follows based on such a basis,

**Step 1**. Setting the ROI for the part of CT medical images: *A*(0, 95), *B*(256, 95), *C*(0, 400), *D*(256, 400), as shown in Fig 5.
**Step 2**. Creating a new image of the same size as the image we want to cut.
**Step 3**. Copying the original image to the new image.
**Step 4**. Releasing the ROI area.

### Discern the gallstone

The quality of features extracted from ROI directly affects the accuracy of the result of recognizing the cholelithiasis. In [19], Krizhevsky proposed a method for classifying image on Imagenet, which greatly improved the performance of those traditional features extracting algorithms [20–21]. In our method, we select the method from Krizhevsky to extract features for our lightweight deep convolutional neural network. We use the lightweight convolutional neural network MobilenetsV2 in the system to discern the gallstone.

After inputting ROI into the lightweight convolution neural network, the forward propagation of the neural network, an end-to-end image with the label of a focus of cholelithiasis is generated directly. Meanwhile, an electronic version of the medical report is generated from the system, including the user’s information, the number and size of gallstones, some medical advice and so on.

## Our deep model

Convolution neural network has been widely applied in the fields of image classification, image segmentation and object detection, etc. Deep learning uses multi-layer computing models to learn abstract data representations and to discover complex structures in big data. At present, this technology has been successfully applied to many kinds of pattern classification problems including computer vision. Computer vision analysis of target motion can be divided into three levels: motion segmentation, target detection; target tracking; action recognition, behavior description. Among them, target detection is not only one of the basic tasks to be solved in the field of computer vision, but also the basic task of video monitoring technology. Due to different forms and frequent occlusion of targets in video, their movement is irregular. Meanwhile, considering the depth of field, resolution, weather, lighting and other conditions of video monitoring and the diversity of scenes, the results of target detection algorithm will directly affect the follow-up tracking, action recognition and behavior description. At present, the main target detection algorithms can be divided into two categories: One-stage target detection algorithm, Two-stage target detection algorithm. One-stage target detection algorithm does not need Region Proposal Stage, and can directly generate the class probability and corner value of objects through one Stage. Typical algorithms include YOLO, SSD and CornerNet. The two-stage target detection algorithm divides the detection problem into two stages. In the first stage, they first produce Proposals that contain approximate location information of the target. In the second stage, classification and location refinement of candidate regions are carried out. Typical representatives of such algorithms include R-cnn, Fast R-cnn, Faster R-cnn, etc. Image classification is the first problem to be solved in computer vision task, which is the basis of object detection, semantic segmentation and other tasks. The traditional methods of image classification are feature description and detection, which may be effective for some simple image classification, but due to the complexity of the actual situation, the traditional classification methods are overwhelmed. At present, the most popular way in image classification task is to use multi-layer depth computing model to learn abstract information in big data. Typical representatives of networks in this category include AlexNet, VGG, GoogLeNet, ResNet.

Although the performances of the the networks have been improved, it is followed by the problem of the storage model and the speed at which the model is predicted. Hundreds of layers of networks have a large number of weight parameters that need to be saved, which requires a lot of memory on the device, particularly hard for mobile devices, like smart phones and so on. In practice, if the model runs at the millisecond level, the efficiency problem we can get convolution neural network out of the lab and more widely used in the mobile devices. The main idea of the lightweight model is to design a more efficient method of network calculation mainly for the convolution, so that the network parameters are reduced and network performance will not be lost. The development of lightweight convolution neural network and mobile terminal neural network model make it possible to deploy a small deep convolution neural network model at the mobile terminal devices.

As a new type of convolutional neural network, lightweight convolutional neural network has the characteristics of small scale and fast speed. The main idea of its design is to design more efficient network computing mode (mainly for convolution mode), so as to reduce network parameters without losing network performance. At present, there are four main directions for designing lightweight neural network models in industry and academia: manually designing lightweight neural network models; automatic design of Neural network based on Neural Architecture Search (NAS); CNN model compression; automatic model compression based on AutoML. The lightweight model used in our system is MobileNetsV2. When designing MobileNet V1, it refers to the traditional chain architecture such as VGGNet to improve the network depth by stacking convolutional layer, so as to improve the identification accuracy. But stacking too many layers of convolution will present a problem, namely varnishing. Resnet makes it easier for information to flow between layers, including reusing features in forward propagation and mitigating the disappearance of gradient signals in back propagation. Therefore, skip connection is added in the improved version of MobileNet V2, and good improvements are made to ResNet and MobileNet V1.

### Depthwise separable convolutions

**Fig 6:**
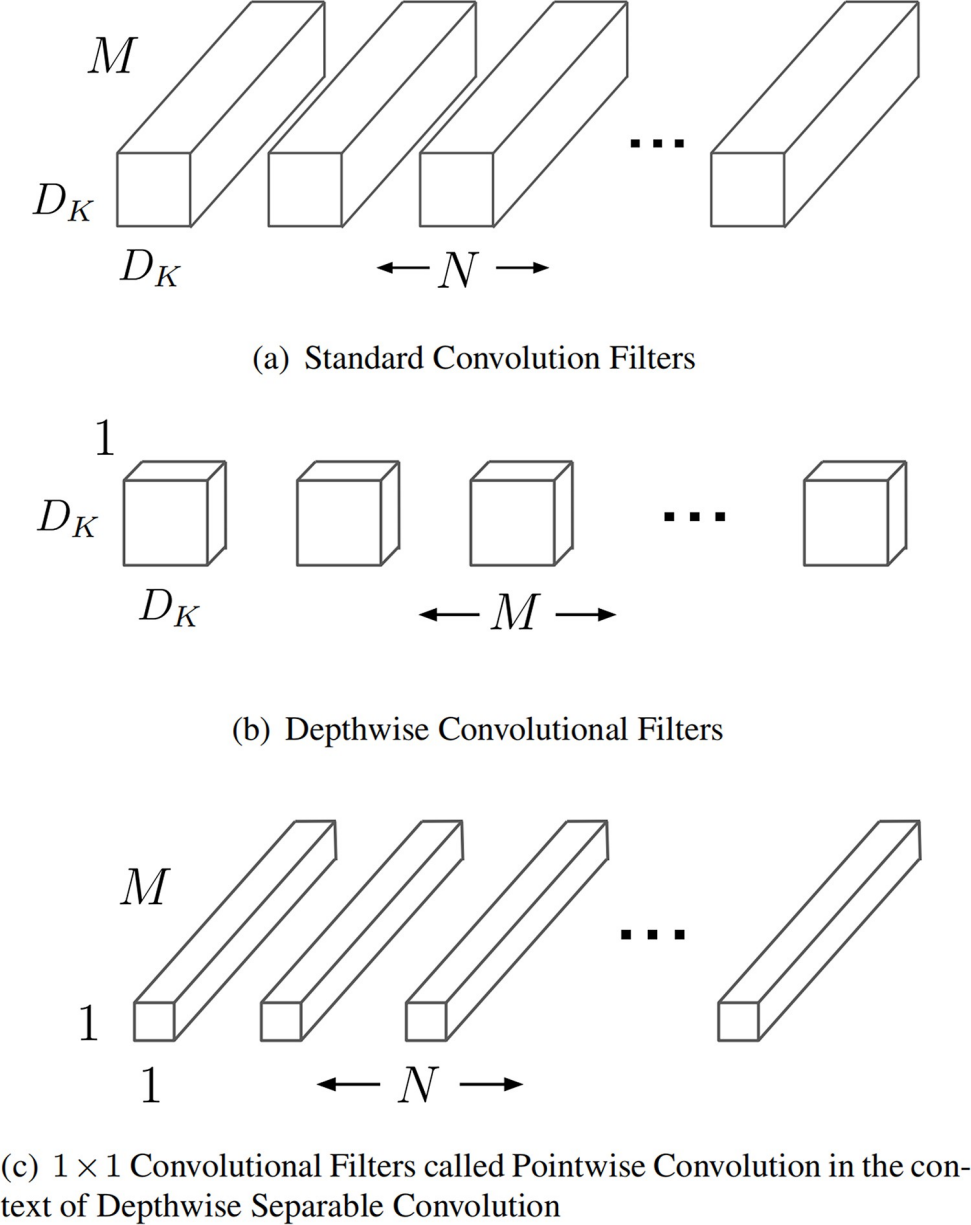
The standard convolutional filters in (a) are replaced by two layers: Depthwise convolution in (b) and pointwise convolution in (c) to build a depthwise separable filter.

MobileNetsV2 is designed on the basis of depthwise separable convolutions, which is a crucial key in the network. This structure is similar to conventional convolution and can be used to extract features. But compared with conventional convolution operation, the number of parameters and operation cost are lower. We can encounter this kind of structure in some lightweight networks. In general, it decomposes the standard convolution into depthwise convolutions and pointwise convolutions, as shown in Fig 6.

Instead of the standard convolution, one of depthwise convolution’s convolution kernel is responsible for a channel of the input picture. And each convolution kernel of a standard convolution operates simultaneously on each channel of the input image. Pointwise convolution is very similar with the standard convolution. The kernel size is 1 × 1 × *M*, and M is the number of channels in the previous layer.

In the same case of *h*_*i*_ × *w*_*i*_ × *d*_*i*_ input tensor *L*_*i*_, standard convolutional layers have the computational cost of *h*_*i*_ ⋅ *w*_*i*_ ⋅ *d*_*i*_ ⋅ *d*_*j*_ ⋅ *k* ⋅ *k* and depthwise separable convolutional layers have the cost of *h*_*i*_ ⋅ *w*_*i*_ ⋅ *d*_*i*_(*k*^2^ + *d*_*j*_). We use *k* = 3 (3 × 3 depthwise separable convolutions) in this paper.

### Linear Bottlenecks

MobileNetV2 besides uses depthwise separable convolution to replace the standard convolution, also made a crucial experiments. In the experiment, the author used the width multiplier parameter to make the model channel reduction, which was equivalent to “thinning” the network model. After the channel is reduced, the feature information can be more concentrated in the reduced channels. However, if a nonlinear activation layer, such as ReLU, is added, there will be a large loss of information, which will affect the learning of the whole network. So in the MobileNetV2 network model, the nonlinear activation layer is not connected after the dimension reduction layer.

For Linear Bottlenecks, we need to pay attention to two aspects. First, for the non-zero value of the output of the ReLU layer, the ReLU layer ACTS as a linear transformation, which can be seen from the curve of ReLU. Second, the ReLU layer can retain the input manifold information, but it is only effective when the input manifold is a low-dimensional subspace of the input space.

### Inverted residuals

In the very deep neural network training work, traditional residual solved the network performance degradation problem which often appeared. Traditional residual can make the feature information in the shallow layer of the deep network be reused in the deep layer, thus alleviating the problem of gradient disappearance and improving the generalization ability of the neural network.

**Fig 7:**
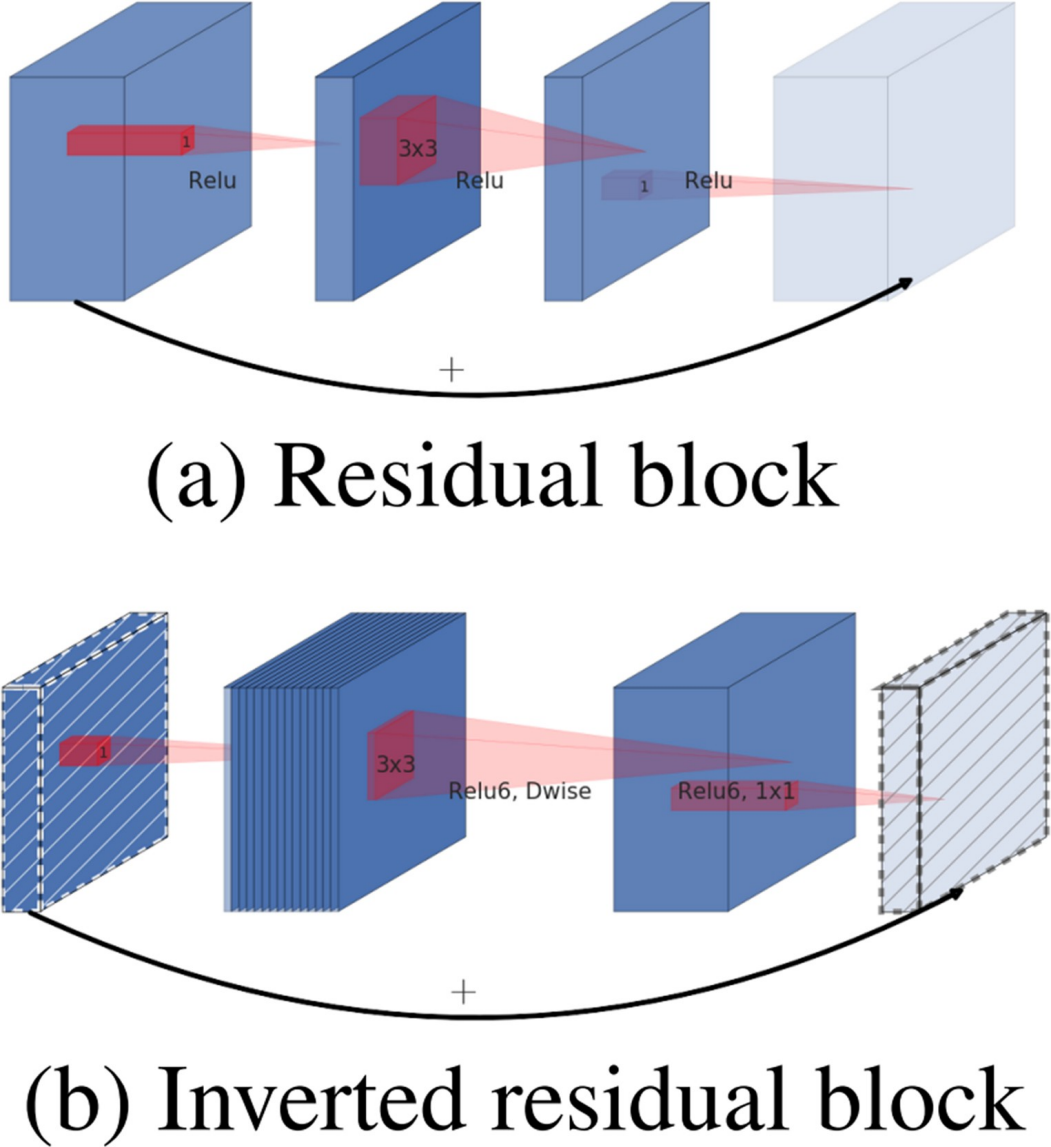
The difference between residual block (a) and inverted residual (b).

In Fig 7, the traditional residual block in the left (a) figure first used 1 × 1 convolution to reduce the dimension of the input feature map, then carried out 3 × 3 convolution operation, and finally used 1 × 1 convolution to increase the dimension. Fig 7 to the right (b) is proposed in this paper the structure, use 1 × 1 first convolution of the input dimension of feature map, and then use 3 × 3 depthwise convolution way do convolution operation, finally using 1 × 1 convolution operation to reduce the dimension. Note that after the convolution operation of 1 × 1, the ReLU activation function is no longer used, but the linear activation function is used to retain more feature information and ensure the expressive ability of the model.

We can see the structure of bottleneck residual block in Table 1. It has the cost of *h* × *w* × *d* × *t*(*d*′ + *k*^2^ + *d*″), with a block of size *h* × *w*, expansion factor *t* and kernel size *k* with *d*′ input channels and *d*″ output channels. We use ReLU6 as the non-linearity because of its robustness when it is used with low-precision computation.

**Table 1:** Bottleneck residual block transforming from *k* to *k*′ channels, with stride *s*, and expansion factor *t*.

Input	Operator	Output
*h* × *w* × *k*	1 × 1 conv2d, ReLU6	*h* × *w* × (*tk*)
*h* × *w* × *tk*	3 × 3 dwise *s* = *s*, ReLU6	$\frac{h}{s}\times \frac{w}{s}\times \left(tk\right)$
$\frac{h}{s}\times \frac{w}{s}\times \text{tk}$	linear 1 × 1 conv2d	$\frac{h}{s}\times \frac{w}{s}\times k^{\prime }$

### MobileNetV2

The architecture of MobileNetV2 is described in detail in the Table 2. We use kernel size 3×3 as standard for modern networks, and utilize dropout and batch normalization in the training.

**Table 2:** MobileNetV2: Each line describes a sequence of 1 or more identical (modulo stride) layers, repeated *n* times, number *c* of output channels, expansion factor *t*, stride *s*.

Input	Operator	t	c	n	s
224^2^×3	conv2d	-	32	1	2
112^2^×32	bottleneck	1	16	1	1
112^2^×16	bottleneck	6	24	2	2
56^2^×24	bottleneck	6	32	3	2
28^2^×32	bottleneck	6	64	4	2
14^2^×64	bottleneck	6	96	3	1
14^2^×96	bottleneck	6	160	3	2
7^2^×160	bottleneck	6	320	1	1
7^2^×320	conv2d 1×1	-	1280	1	1
7^2^×1280	avgpool 7×7	-	-	1	-
1×1×1280	conv2d 1×1	-	k	-	

In Table 3, we compare the needed sizes for each resolution between MobileNetV1, MobileNetV2 and ShuffleNet. ShuffleNet is a classic lightweight convolution neural network, it uses the pointwise group convolution to reduce the computational complexity, thus solves the problem that 1×1 convolution requires a lot of computing resources.

**Table 3:** The compared sizes for each resolution between MobileNetV1, MobileNetV2 and ShuffleNet.

Size	MobileNetV1	MobileNetV2	ShuffleNet(2x,g = 3)
112×112	1/O(1)	1/O(1)	1/O(1)
56×56	128/800	32/200	48/300
28×28	256/400	64/100	400/3600K
14×14	512/200	160/62	800/310
7×7	1024/199	320/32	1600/156
1×1	1024/2	1280/2	1600/3
max	800K	200K	600K

In the SSD prediction layer, we introduce a mobile-friendly regular SSD variant that replaces all regular convolution with separable convolution. This design is consistent with the overall design of Mobilenet and is considered to be more computational efficient. It is called the modified version SSDLite. It is shown in Table 4, that SSDLite dramatically reduces both parameter count and computational cost comparing with regular SSD.

**Table 4:** Comparison of the size and the computational cost between SSD and SSDLite configured with MobileNetV2.

Network	Params	MAdds
SSD	14.8M	1.25B
SSDLite	2.1M	0.35B

## Implement the system

The implementation of intelligent diagnostic system for cholelithiasis is based on Android 6.0 system. The software of programming used is Android Studio 2.2, and the OpenCV [22] 2.4.10 is used for processing image. The main programming languages are Java and C++. The platform of programming is a 64-bit Windows 7 system. The platform for testing includes Android virtual machine built-in Android Studio2.2, Xiao Mi 7, HTC One M8.

### The user interface

The user interface includes inputting information and recognize the cholelithiasis. Inputting information includes inputting user’s basic information and uploading the CT image to the system. The user’s basic information is input by the user manually. Meanwhile, the users needs to upload manually his own CT image to the system. The workflow of the system is as follows:

1. Inputting user’s basic information such as the user’s name, gender, height, weight and so on.
2. Querying whether the user’s diagnostic record exists in the database. If it exists, the last diagnostic record is displayed to the user; if not, the next step is to recognize the cholelithiasis.
3. Waiting for the result of the processing and outputting the diagnostic result from the system. At the same time, generating an electronic medical report from the system.
4. Saving the user’s diagnostic record into the database.

### Implement the algorithm

Android Studio 2.2 supports that using the Cmake to compile C++ code for interaction so that the user interface is implemented by using C++ code. Here, using C++ has three advantages over Python: We apply Python in the early training of neural network so that we directly call the Python code that has been trained to complete the system in the implementation of algorithm part. In order to unify the format of some image data of Python and C++, we use OpenCV as the bridge for representation of image and uses the MAT defined by OpenCV as the main format for processing image.

SQLite is used as the database platform by considering the data volume size and operation platform of the actual application. In order to improve the efficiency of operating data, the user’s diagnostic record is stored in the form of an eigenvector, which can greatly reduce the storage space of diagnostic record. The efficiency of the system of reading diagnostic records can be greatly improved.

## Data experiments

The types and forms of cholelithiasis are diverse, and the lesions of some cholelithiasis are also very similar, which greatly hinders the correct diagnosis and treatment of cholelithiasis. In cholelithiasis, doctors observe the medical images such as CT images and B-Mode Ultrasound Image to determine whether a patient has cholelithiasis and determine the location and type of gallstones. The CT images or computed tomography, uses collimated physical rays such as x-rays, *γ*-rays and ultrasonic waves to scan a part of the body with highly sensitive detectors, and ultimately output the CT images. CT images have the characteristics of high diagnostic value and universal application, especially in the diagnosis of gallbladder diseases so that we use CT images as the dataset and the resolution of the image is 512 × 512.

**Fig 8:**
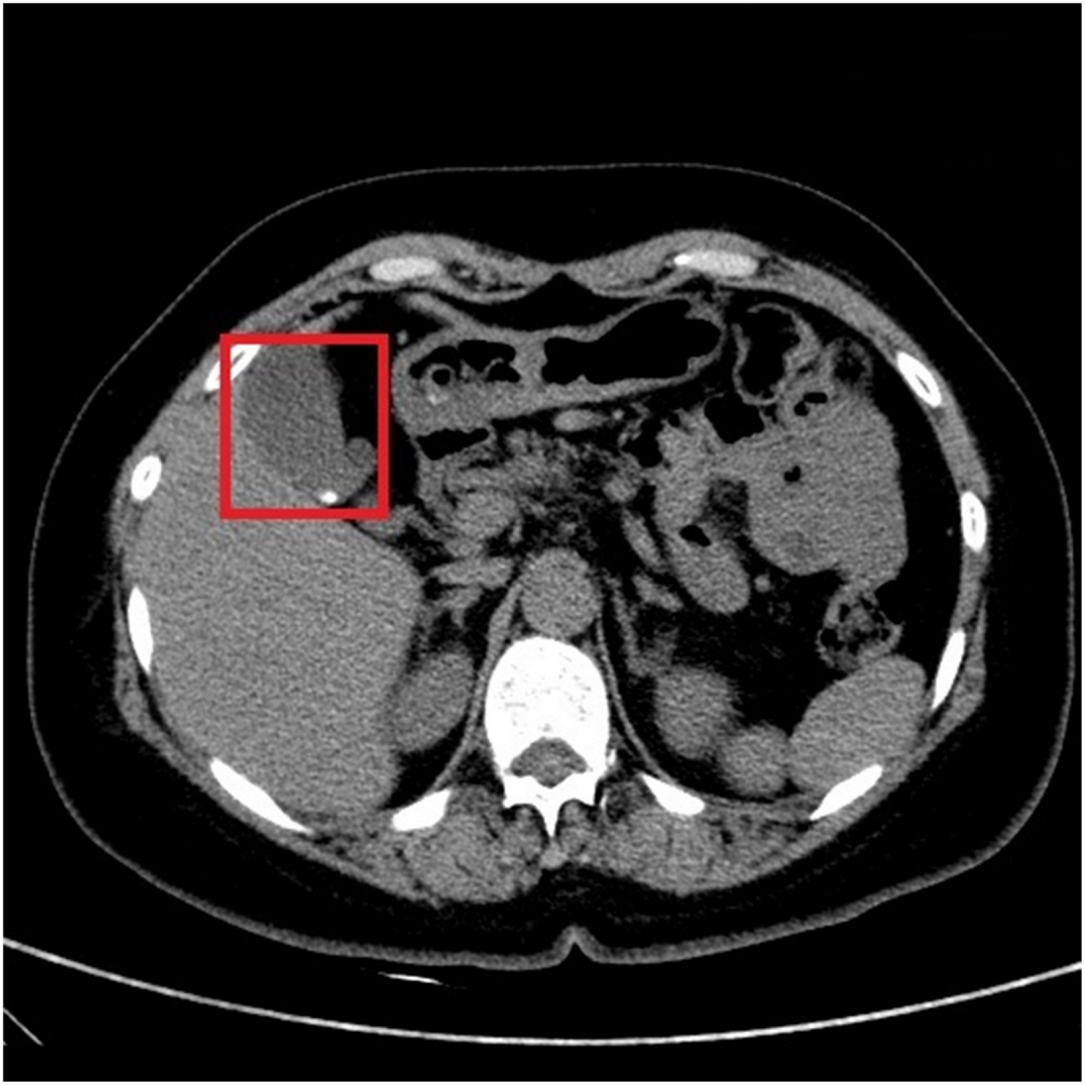
The CT image of a person has a lower degree of disease.

**Fig 9:**
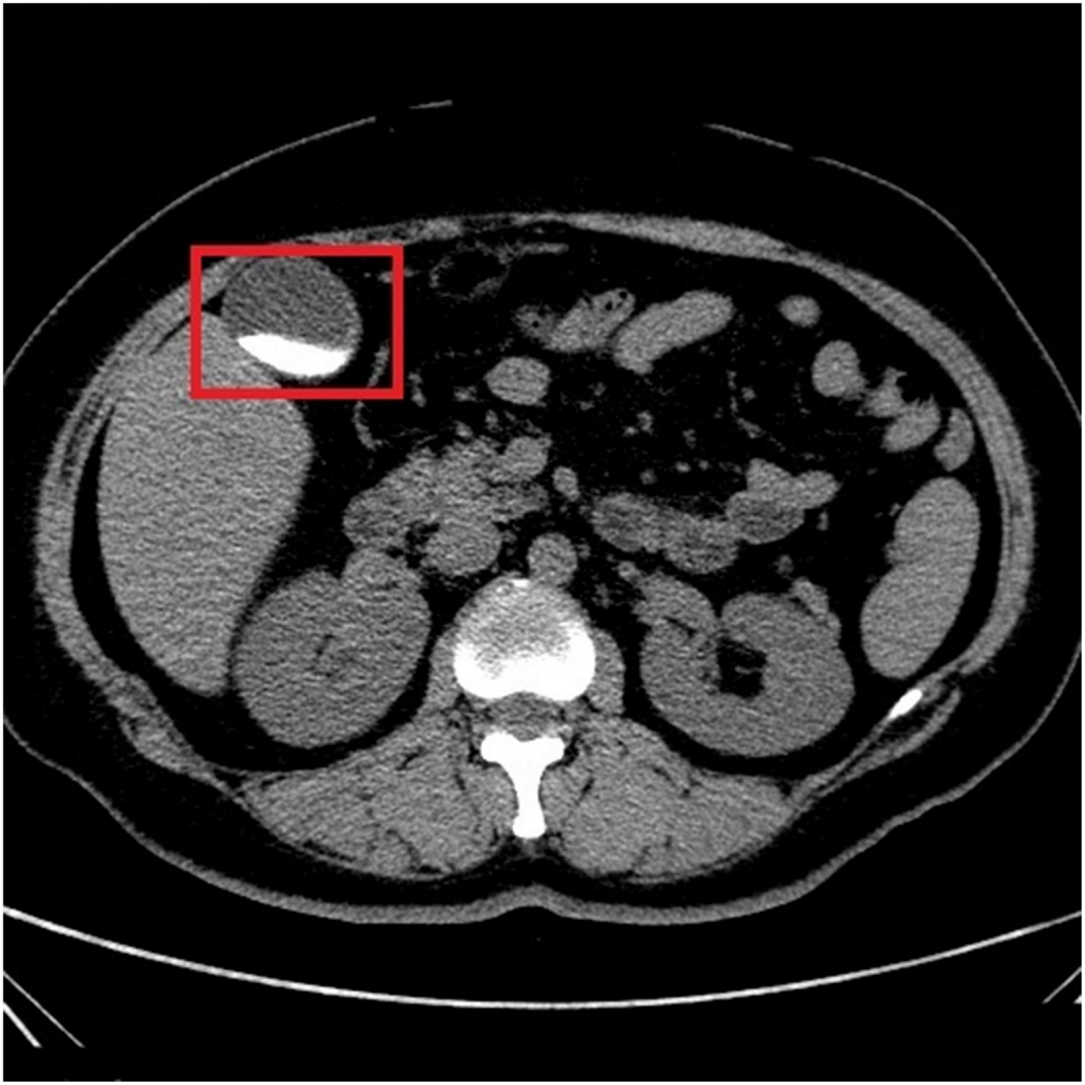
The CT image of a person has a higher degree of disease.

Doctors need to observe the CT images of cholelithiasis to determine where the gallstones are located and determine the level of the disease. In Figs 2 and 3, the gallbladder of healthy person is all light gray color, and gallstones are in white and differ significantly from the gallbladder in the CT images. We attempt to determine the level of disease also by observing the form of gallstones. For instance, the gallstone in Fig 8 is relatively small, so we can determine that the patient has cholelithiasis, but the grade of disease is in low level. On the contrary, the gallstone in Fig 9 is relatively large, indicating that the patient has a higher degree of disease.

We collect data set of cholelithiasis from the Third Provincial Hospital of Shandong Province, China. The permission was obtained from patients for their data to be used in this research. It contains CT images of 100 patients with cholelithiasis. After revolving the images, we obtain in total 1300 CT images of cholelithiasis, and 673 CT images are randomly selected for training and the rest 627 images are used for verification.

### Experimental results

**Fig 10:**
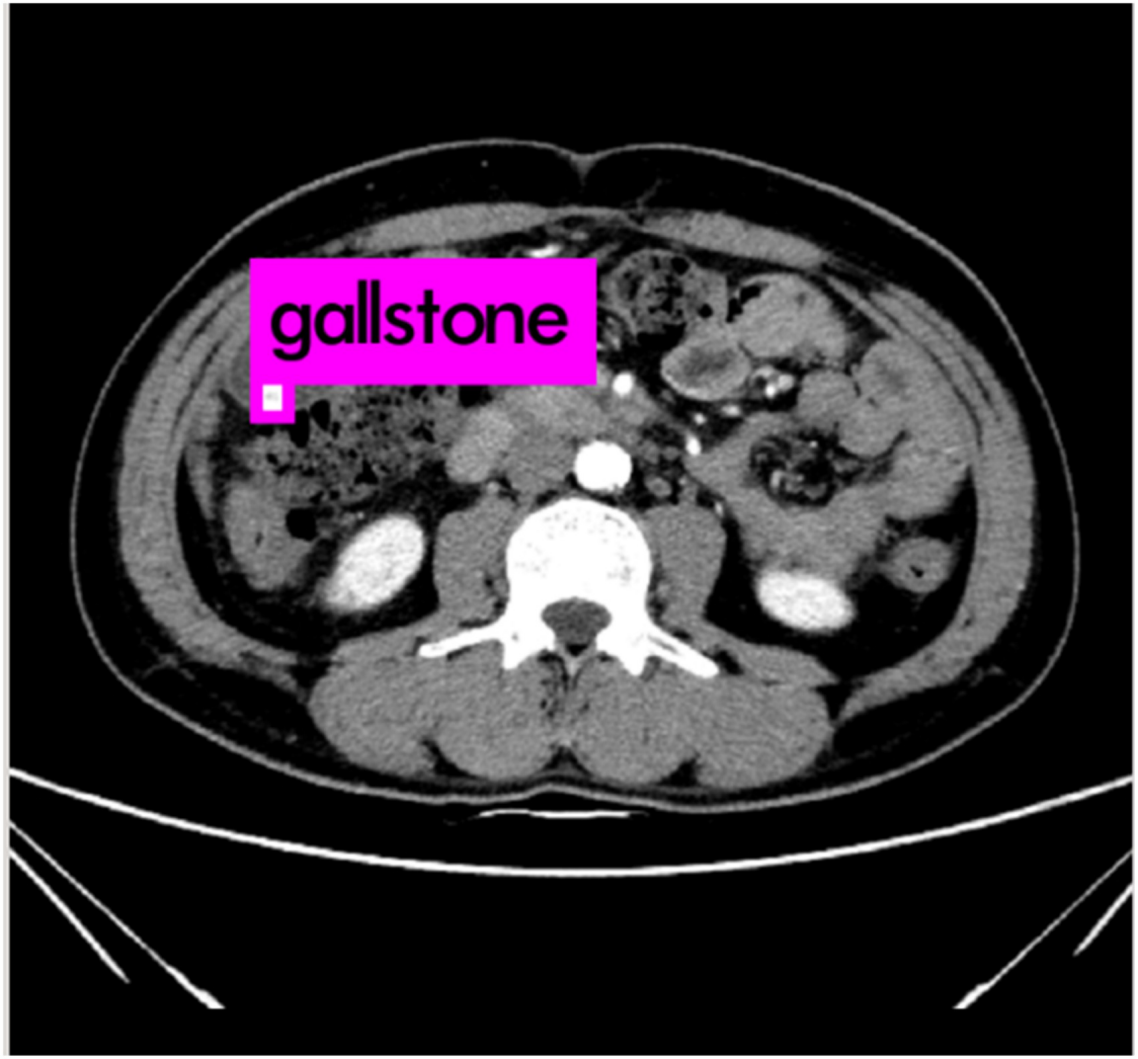
The CT image with the lesions discerned.

Our systems can detect the presence of gallstones in the CT images, determining and marking its location and size to assist doctors in diagnosing diseases ulteriorly, as shown in Fig 10. Also, our system can discern different types of gallstones such as granulated stone and muddy stone. Some important organs that include liver, gall and spine can also be discerned by our system. We evaluate and compare the performance of MobileNetV2 and MobileNetV1 as feature extractors for object detection with a modified version of the Single Shot Detector (SSD) on dataset in Table 5. We also compare the performance of our system with YOLOv2 from and original SSD(with VGG-16 from.

**Table 5:** Performance comparison of MobileNetV2 + SSDLite and other realtime detectors on the cholelithiasis dataset.

Network	Accuracy/%	Params	MAdds
SSD300	92.0	36.1M	35.2B
SSD512	91.8	36.1M	99.5B
YOLOv2	90.7	50.7M	17.5B
MobileNetV1+SSDLite	92.4	5.1M	1.3B
MobileNetV2+SSDLite	91.0	4.3M	0.8B

According to the practical requirement of the intelligent diagnostic system for cholelithiasis, the experiment is mainly divided into two parts:

1. Verifying the recognition accuracy of the system for gallstone disease;
2. Testing the usability of the system, including the run time, energy consumption, UE and so on.

**Fig 11:**
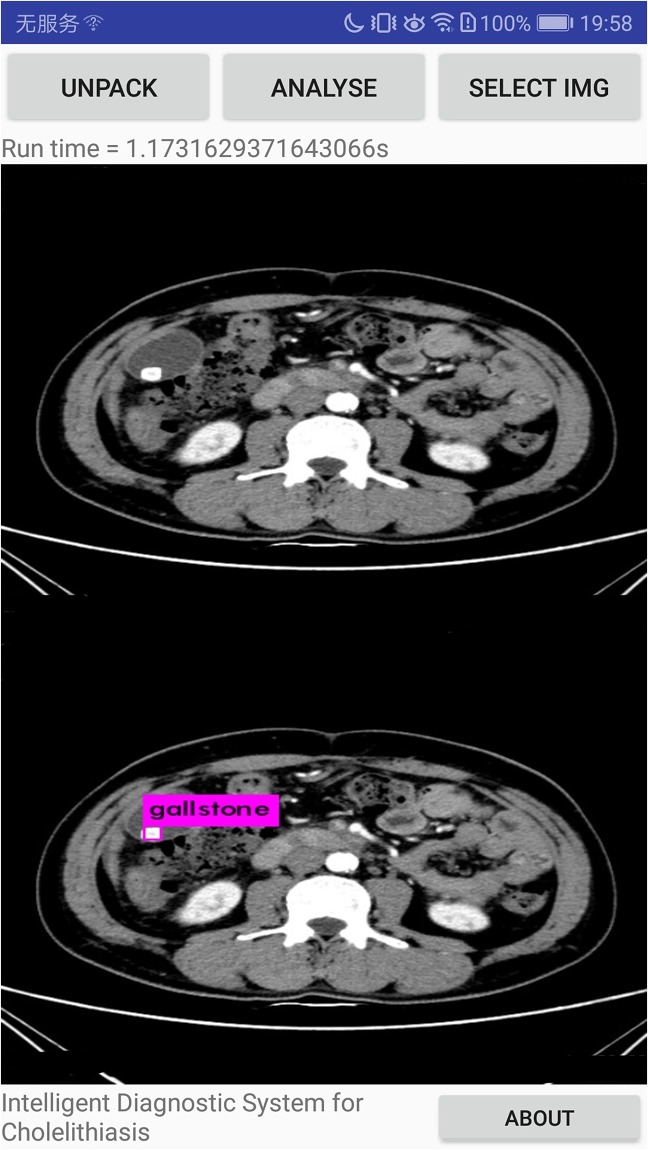
The experimental results on the devices.

**Fig 12:**
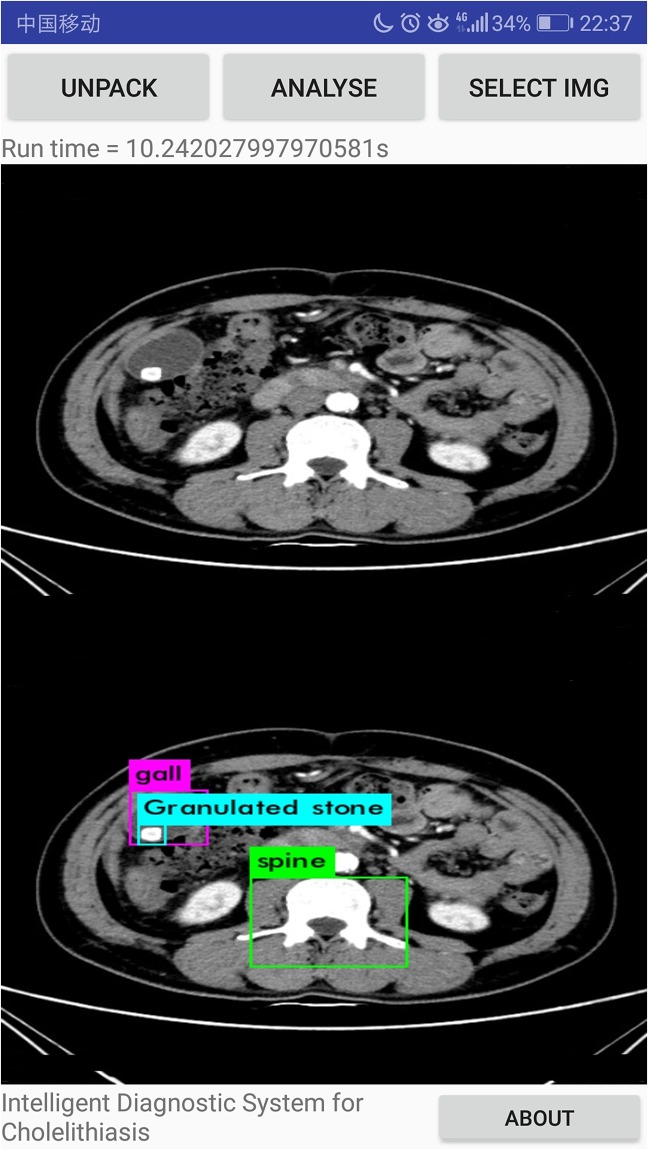
The experimental results that granulated stone is discerned.

**Fig 13:**
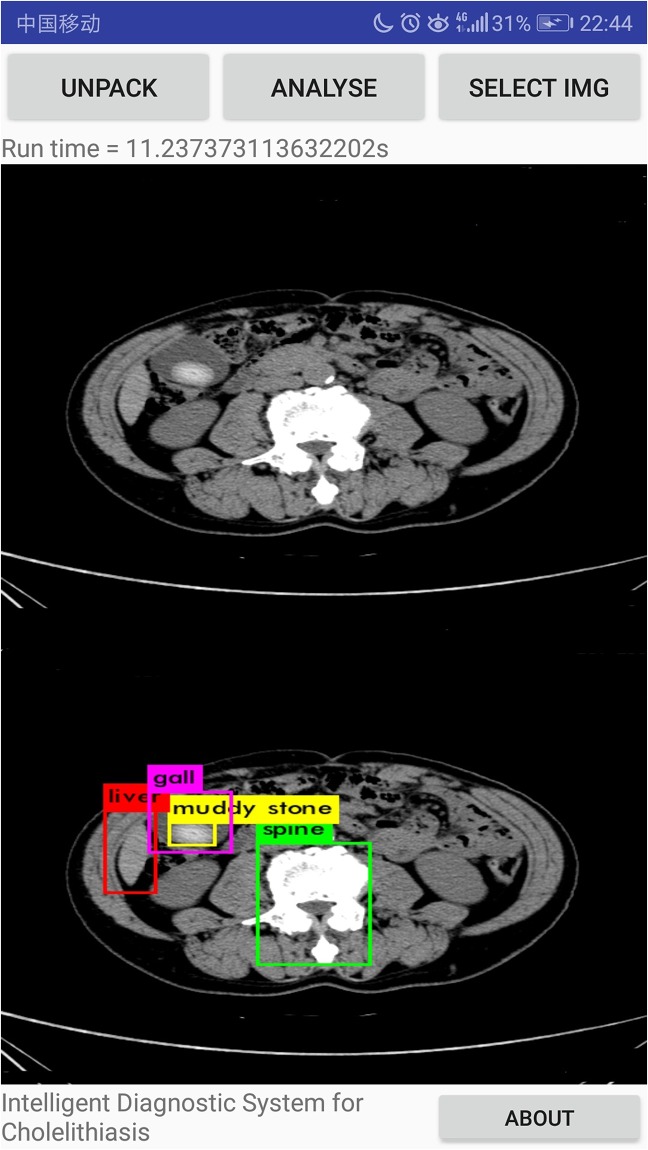
The experimental results that muddy stone is discerned.

According to the final experimental results, 621 CT images of cholelithiasis are successfully discerned by the system, and the lesion location of cholelithiasis is labelled. The accuracy is 92.3%, basically meeting the needs of industrial application. In order to test the system’s accuracy and efficiency in actual use, the system is packaged as apk and installed on different android phones. CT images of new patients with cholelithiasis are collected as a verification set to test. A total of 216 medical CT images of 45 patients with cholelithiasis are recollected to as a validation set in the specific experiment. By revolting, 627 images are collected for verification. The copy the verification set into the phone memory and change the function of interface to call the phone album for discerning gallstone. Specific experimental results are shown in Table 6 and Fig 11. Furthermore, our system can discern the granulated stone and muddy stone such as Figs 12 and 13. And we can see that liver, gall and spine can also be discerned.

**Table 6:** Experimental results on different devices.

Platform	Accuracy/%	Average time/s
Virtual machine	91.0	3.30
Xiao Mi 7	91.1	1.17
Htc One M8	90.8	1.22

Experimental results show that the system can discern the gallstone disease quickly and efficiently with high accuracy, and it also is able to adapt to the effects of different working environment. The actual accuracy is a little lower than experimental results from the system installed on the workbench. The reason is that the operating environment of the system on the workbench is quite different from the operating environment of the system on Android mobile phones. When the system is running on the workbench, there are sufficient memory resources and efficient hardware devices such as GPU in the workbench, making the experimental results more accurate. From data experiments, it is shown that our system has a strong mobility.

## Conclusions

In this work, we developed an AI diagnostic system for cholelithiasis recognition on mobile Android devices. Technically, histogram equalization is used to preprocess the image, and then lightweight convolutional neural network is used to extract cholelith features and recognize cholelithiasis. Experiments show that the system can quickly, in less than 4 seconds, complete the recognition process of cholelithiasis on the premise with average accuracy rate around 90.8%.

In the design and implementation of the system, the accuracy and efficiency are taken into account. As well, features of cholelith can be extracted by the method of deep learning. Compared with the same type of application at home and abroad, the process of image recognition is deployed on the mobile side for the first time. The system is independent on the network transmission, and the entire device is the same size as a pocket, which is portable and stable. The need for diagnose the cholelithiasis intelligently can be well achieved in a portable and mobile environment. We also get important inspiration in [23–36].

## Supporting information

S1 Fig CT image 1. (TIF)

S2 Fig CT image 2. (TIF)

S3 Fig CT image 3. (TIF)

S4 Fig CT image 4. (TIF)

S5 Fig CT image 5. (TIF)

S6 Fig CT image 6. (TIF)
