# Supplementary figures and images for: An artificial intelligent diagnostic system on mobile Android terminals for cholelithiasis by lightweight convolutional neural network
Source: PLoS One. 2019 Sep 12;14(9):e0221720. doi: 10.1371/journal.pone.0221720 (PMC6742400; doi:10.1371/journal.pone.0221720)

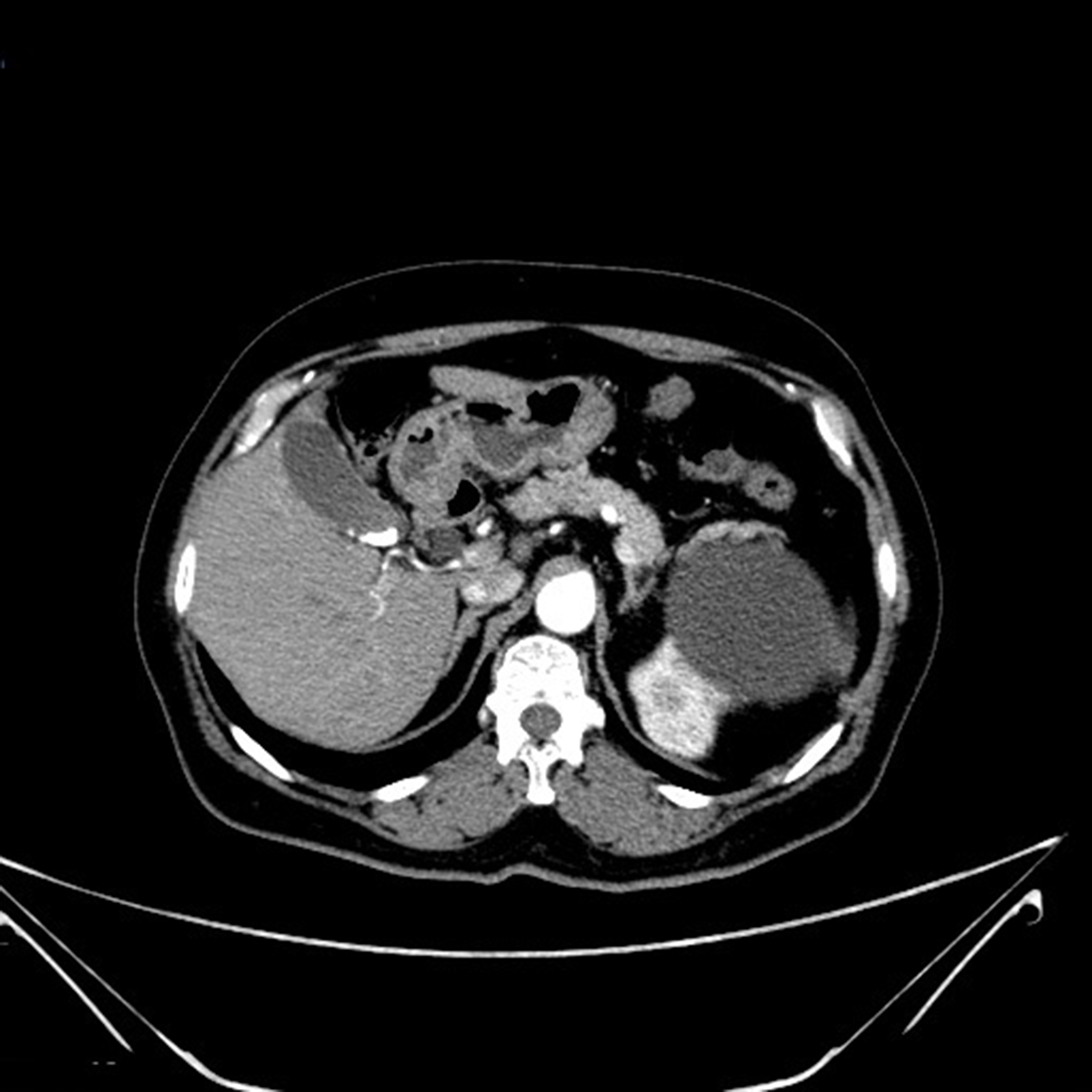

Supplement: S1 Fig — (TIF) [file pone.0221720.s001.tif]

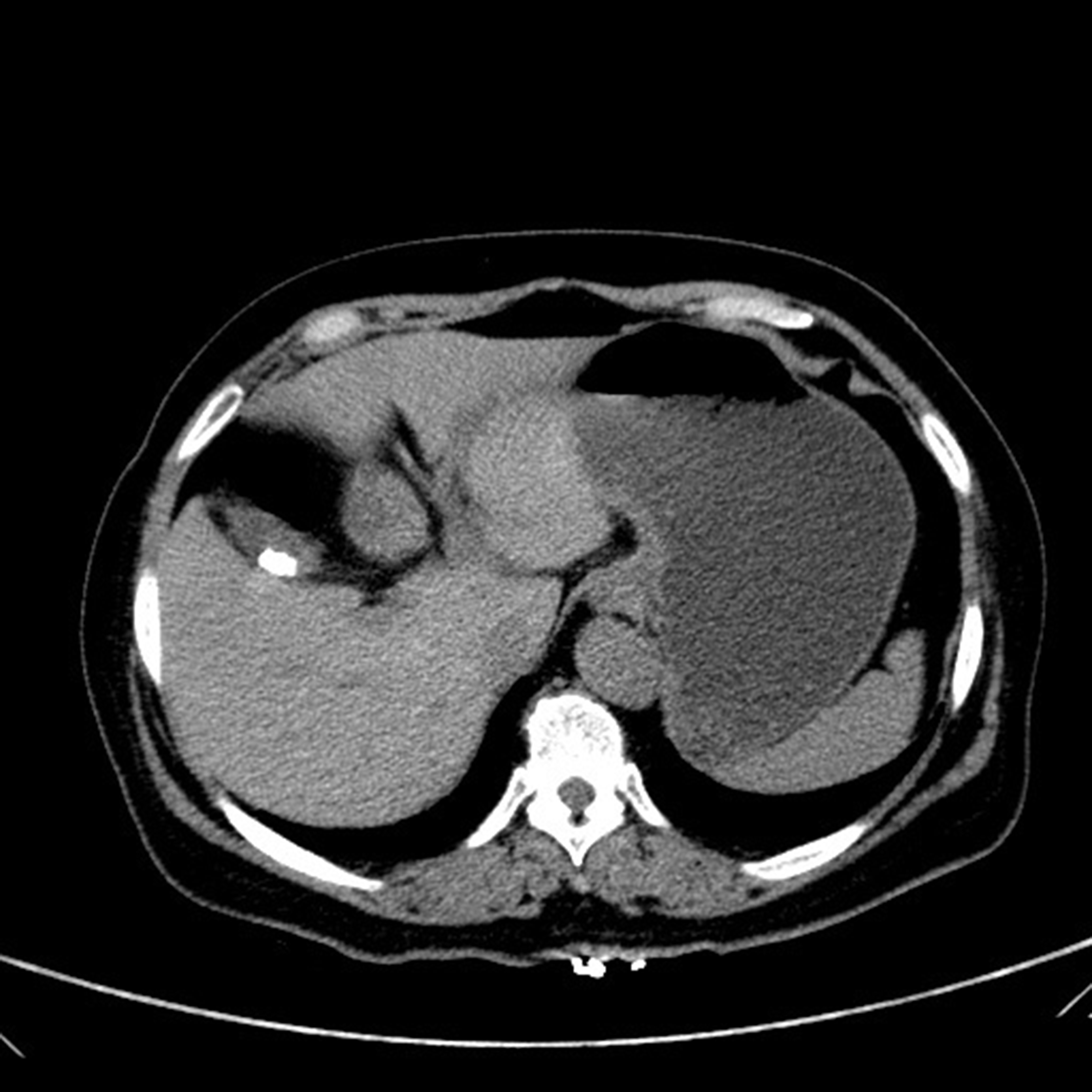

Supplement: S2 Fig — (TIF) [file pone.0221720.s002.tif]

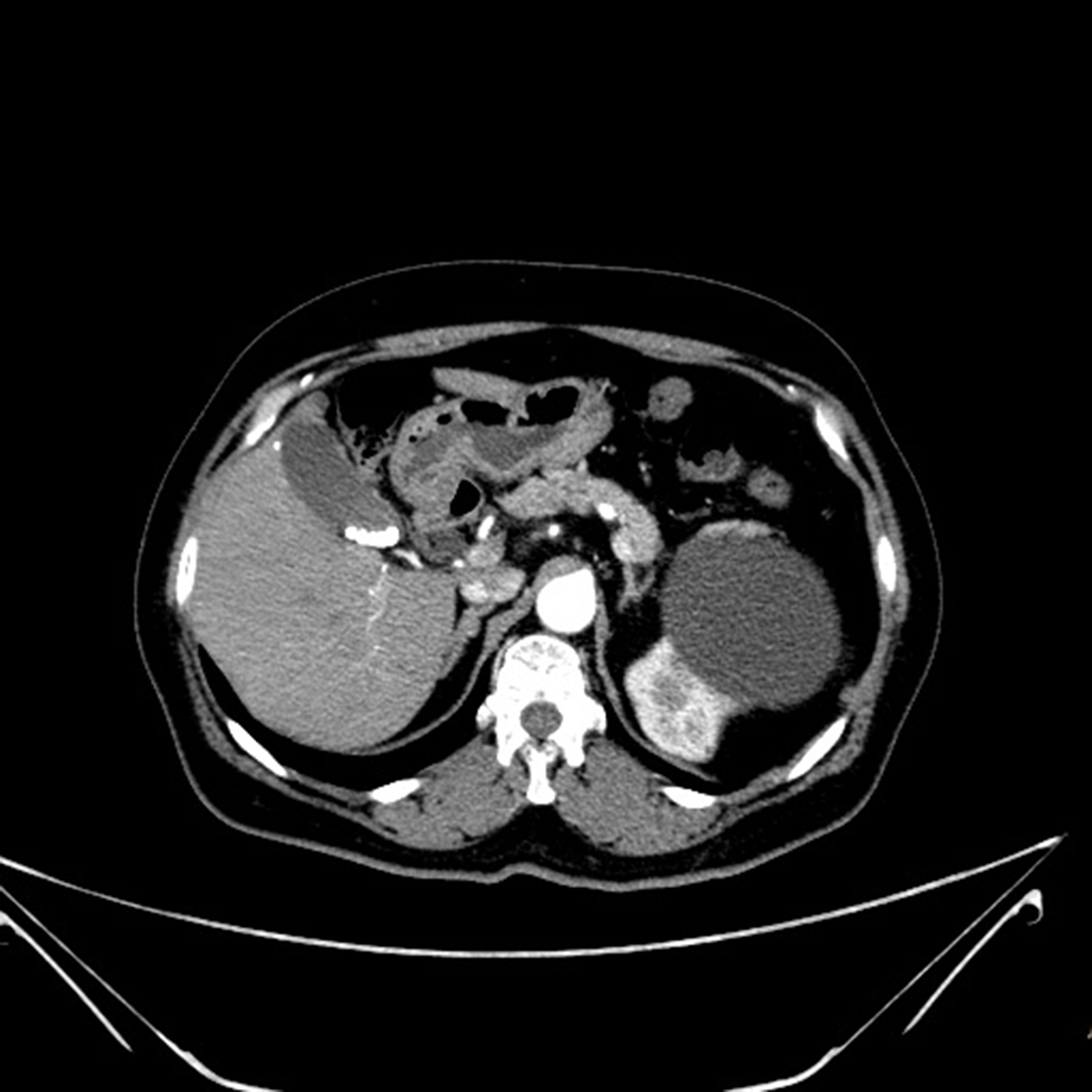

Supplement: S3 Fig — (TIF) [file pone.0221720.s003.tif]

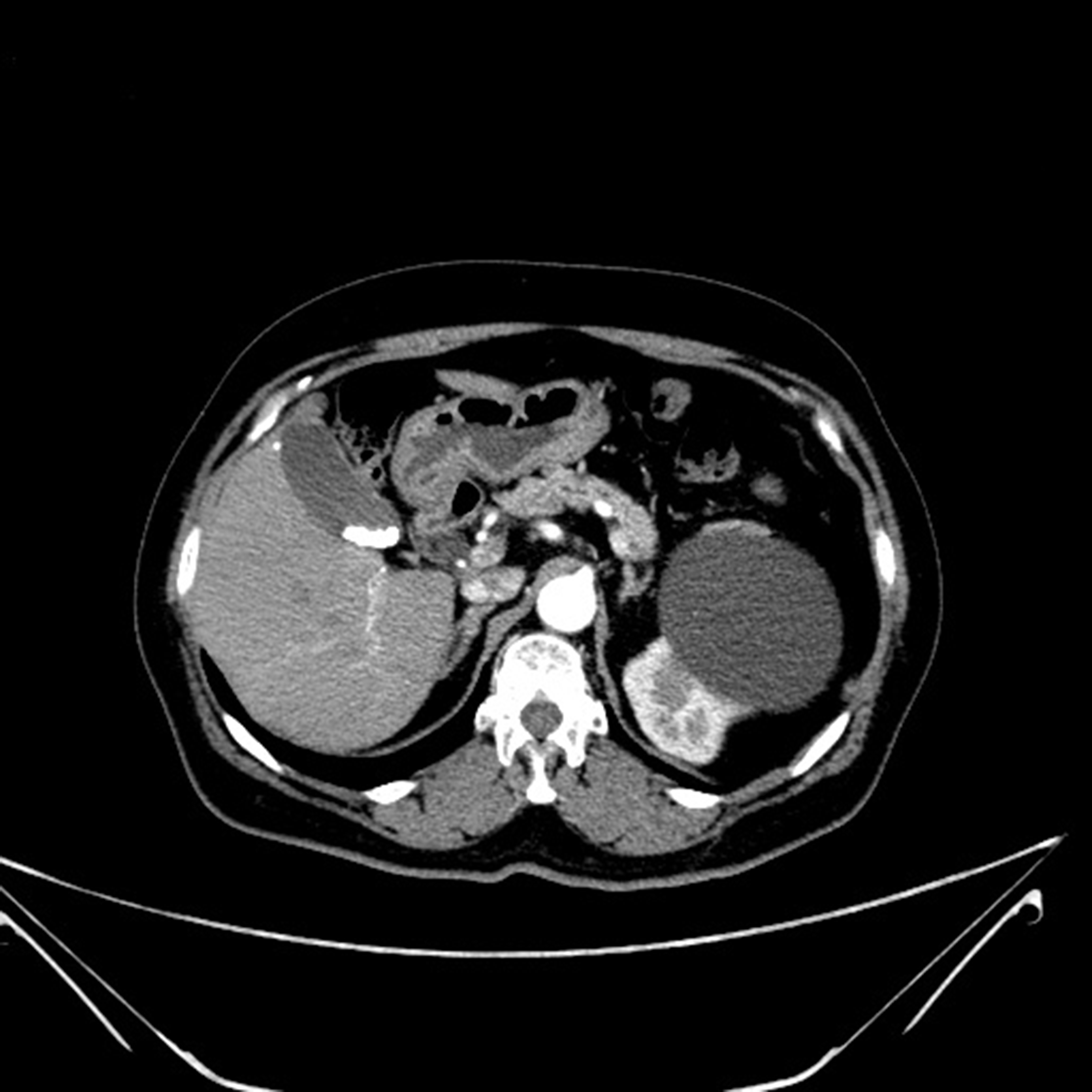

Supplement: S4 Fig — (TIF) [file pone.0221720.s004.tif]

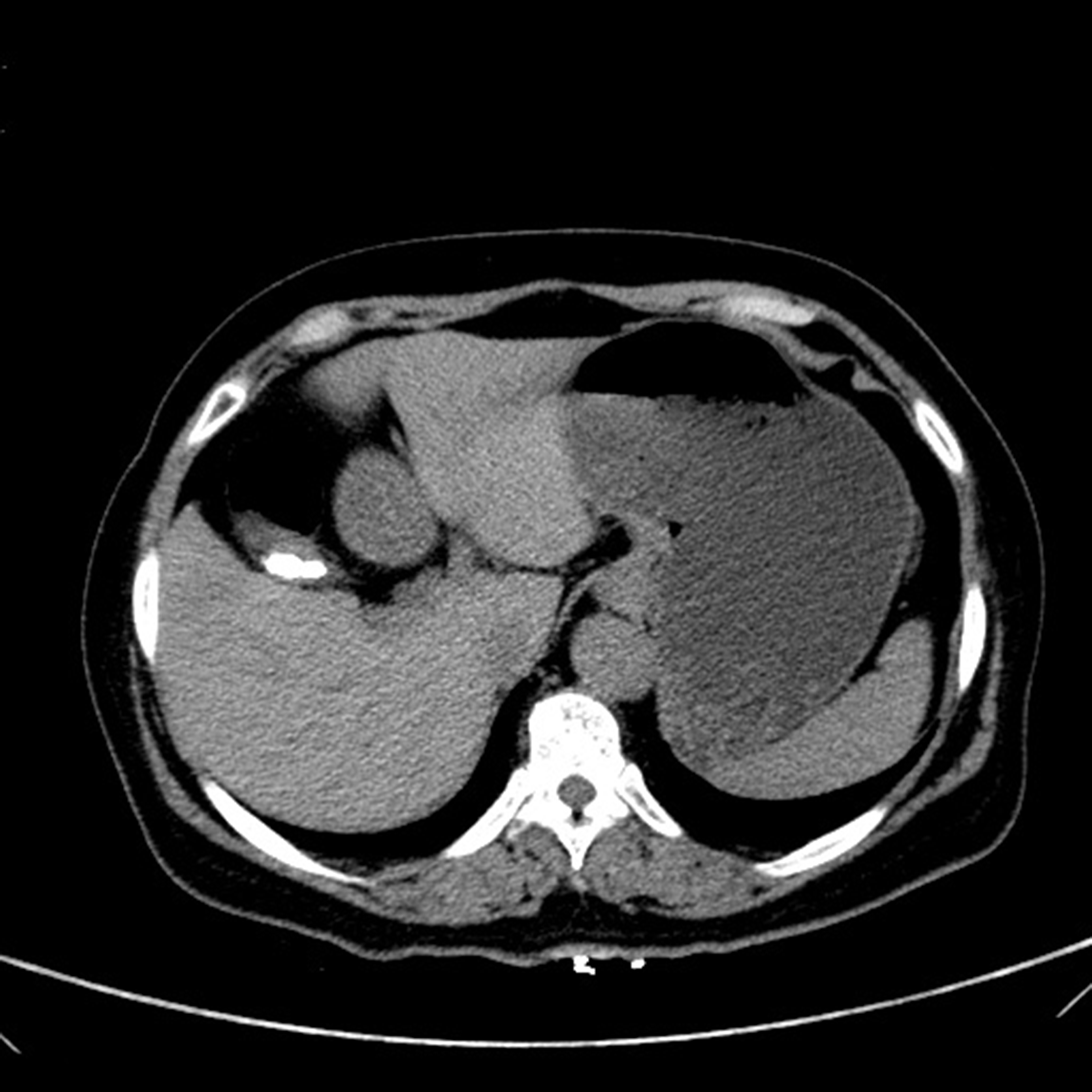

Supplement: S5 Fig — (TIF) [file pone.0221720.s005.tif]

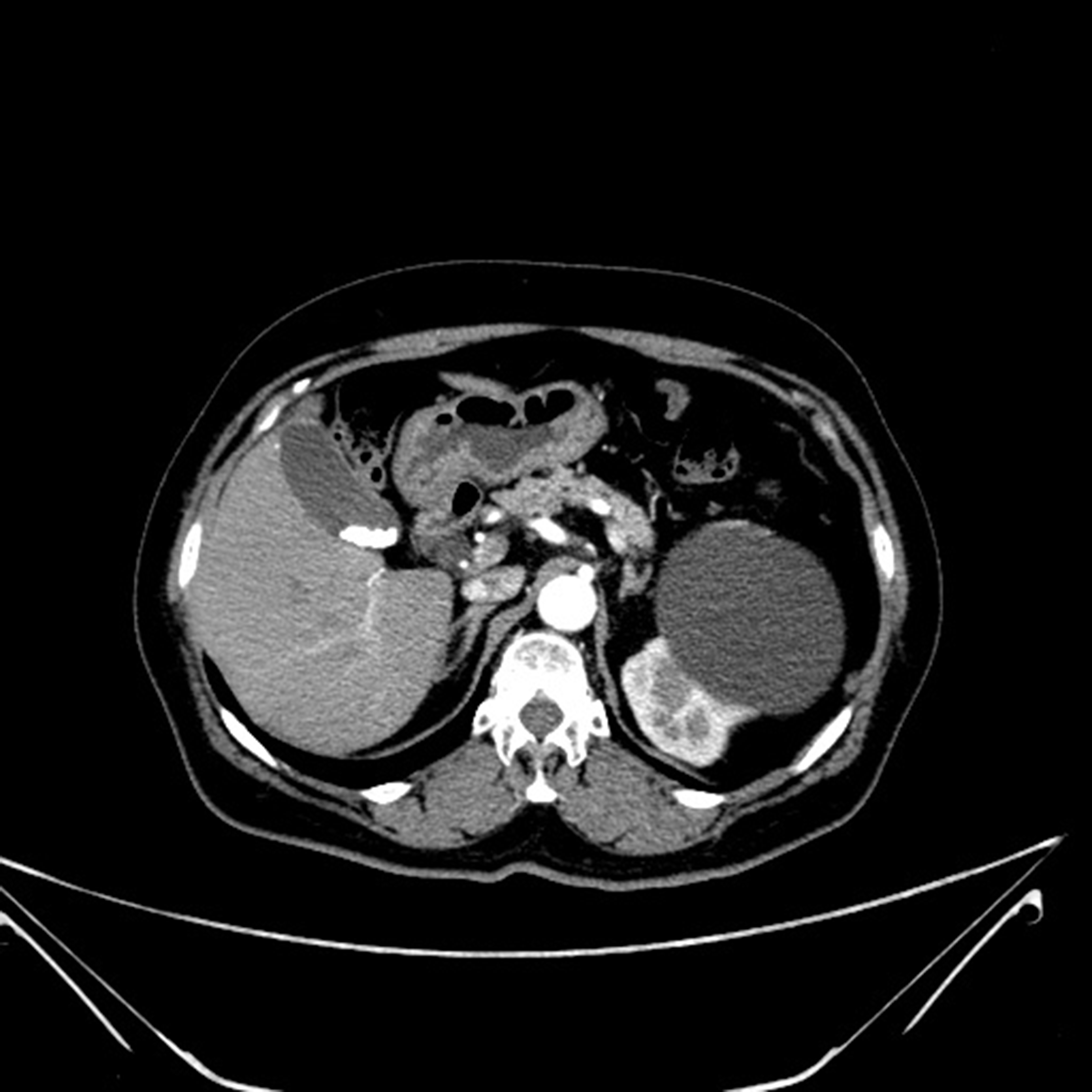

Supplement: S6 Fig — (TIF) [file pone.0221720.s006.tif]
